# Fast-spiking parvalbumin-positive interneurons in brain physiology and Alzheimer’s disease

**DOI:** 10.1038/s41380-023-02168-y

**Published:** 2023-07-07

**Authors:** Sara Hijazi, August B. Smit, Ronald E. van Kesteren

**Affiliations:** 1 Department of Pharmacology, University of Oxford, Oxford, OX1 3QT UK; 2 Department of Molecular and Cellular Neurobiology, Center for Neurogenomics and Cognitive Research, Amsterdam Neuroscience, Vrije Universiteit Amsterdam, 1081 HV Amsterdam, The Netherlands

**Keywords:** Neuroscience, Physiology

## Abstract

Fast-spiking parvalbumin (PV) interneurons are inhibitory interneurons with unique morphological and functional properties that allow them to precisely control local circuitry, brain networks and memory processing. Since the discovery in 1987 that PV is expressed in a subset of fast-spiking GABAergic inhibitory neurons, our knowledge of the complex molecular and physiological properties of these cells has been expanding. In this review, we highlight the specific properties of PV neurons that allow them to fire at high frequency and with high reliability, enabling them to control network oscillations and shape the encoding, consolidation and retrieval of memories. We next discuss multiple studies reporting PV neuron impairment as a critical step in neuronal network dysfunction and cognitive decline in mouse models of Alzheimer’s disease (AD). Finally, we propose potential mechanisms underlying PV neuron dysfunction in AD and we argue that early changes in PV neuron activity could be a causal step in AD-associated network and memory impairment and a significant contributor to disease pathogenesis.

## Introduction

Parvalbumin-positive (PV) interneurons are GABAergic neurons that are found throughout the brain and have many distinctive morphological and physiological characteristics. Some PV interneurons have the unique ability to fire at high frequencies. Fast-spiking PV neurons are characterized by their inhibitory feedback and feedforward perisomatic projections onto pyramidal neurons, which allow them to control network synchrony and regulate theta and gamma oscillations that are crucial for learning and memory. Consequently, dysfunctional PV neurons have been associated with several disorders that involve network alterations and cognitive impairment, including Alzheimer’s disease (AD). In this review, we present recent evidence pointing to PV interneuron dysfunction as a key pathogenic mechanism in AD and AD-associated memory impairment. First, we highlight the unique properties of PV interneurons that make them indispensable for the proper control of network dynamics and the generation of functional network oscillations. Next, we focus on the importance of PV interneurons in learning and memory. Finally, we discuss how PV interneuron impairments lead to network abnormalities and memory deficits in AD.

## Pv neuron properties

### Fast-signaling properties

PV-expressing neurons can be divided to several subpopulations based on morphology and gene expression, including basket cells, axo-axonic cells, bistratified cells, and some oriens-alveus–lacunosum-moleculare (OLM) cells [1]. Moreover, regional differences have been reported in terms of PV-expressing neurons. In the dentate gyrus, for instance, bistratified cells do not express PV and in the prefrontal cortex, some axo-axonic cells are PV-negative [2]. Here, we will review studies that have investigated fast-spiking PV interneurons, which are named as such because of their distinct ability to fire at high frequencies, without going in much detail about the specific subtype of PV neuron that was studied, as this is often not reported.

**Fig. 1:**
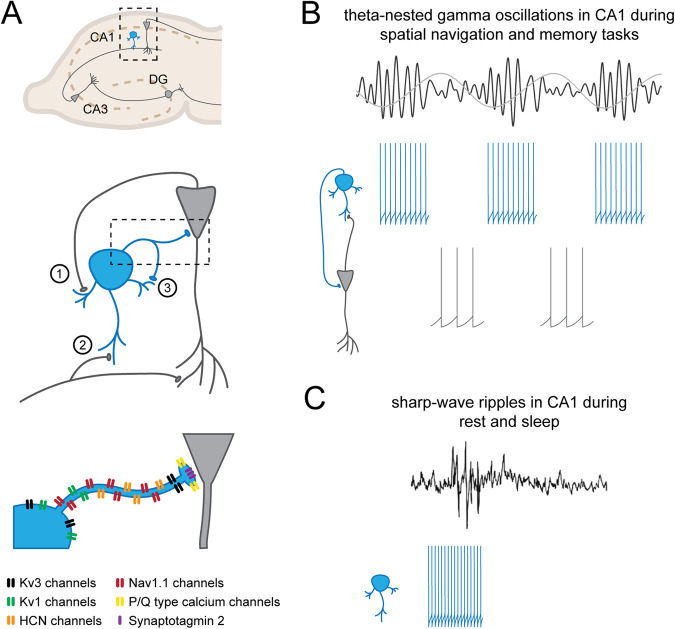
PV neurons control network oscillations. **A** PV neurons (blue) in the CA1 region of the hippocampus control network activity via feedback inhibition (1), feedforward inhibition (2) and autaptic self-inhibition (3). Their fast-spiking properties and fast GABA release onto pyramidal neurons (gray) are due to the expression of specific ion channels and calcium sensors. Nav1.1 sodium and Kv1 and Kv3 potassium channel subunits enable high frequency firing of PV neuron in response to depolarizing currents while HCN channels facilitate action potential propagation. P/Q-type calcium channel subunits and synaptotagmin 2 allow for precise GABA release resulting in accurately timed inhibitory postsynaptic currents in pyramidal neurons. **B** CA1 PV and pyramidal neuron spikes are time-locked with theta-nested gamma oscillations during learning and memory. The oscillatory wave depends on PV neurons firing periodically at gamma frequencies in the trough of the theta wave followed by pyramidal neuron firing at the peak of the theta wave. **C** During rest and sleep, high frequency PV neuron firing is phase-coupled to the oscillatory cycle of sharp-wave ripples oscillations.

Due to a remarkably short action potential (AP) duration and refractory period, fast-spiking PV neurons are capable of generating high-frequency trains of APs when artificially stimulated as well as in freely behaving animals [2, 3]. Recordings from PV neurons in brain slices have shown that APs are initiated close to the soma and propagate with high reliability [4]. High frequency AP firing is followed by extremely fast GABA release in the order of a few milliseconds [5], at PV interneuron axon terminals, which is critical for the proper temporal control of microcircuits [5]. These unique firing and signaling properties depend on the expression of specific ion channels and their distinct subcellular localization (Fig. 1A, for an outstanding review on the molecular, cellular, and network properties of PV interneurons see [2]). The Nav1.1 voltage-gated sodium channel subunit is mainly expressed on axons of fast-spiking PV neurons, allowing for rapid AP propagation and enabling high-frequency firing with very little propagation failure [4]. Other key players in PV neuron firing are Kv1 and Kv3 voltage-gated potassium channel subunits [6]. Kv3 channels, expressed predominantly in fast-spiking PV neurons, have a high activation threshold and rapid deactivation kinetics, resulting in fast repolarization and short AP duration [7]. The short duration of the AP and the fast deactivation of Kv3 channels after spike repolarization enable rapid recovery following each AP, thus contributing to the fast-spiking profile [2, 7, 8]. Kv3 channels are found at the soma of PV neurons, but are also highly expressed at axon terminals, facilitating fast GABA release at synaptic sites [7, 9, 10]. Kv1 channels, on the other hand, are present in the axon initial segment and soma of PV neurons, and are responsible for controlling AP firing threshold and are necessary for a persistent potentiation of PV neuron excitability [6, 11]. A more recent study has confirmed the role for the hyperpolarization-activated cyclic nucleotide-gated (HCN) channels in fast AP signaling [12, 13]. In addition to modulating neuronal excitability, this study revealed that HCN channels enhance AP initiation during sustained firing and facilitate the propagation of APs [12]. HCN channels are activated by hyperpolarization and are permeable to K^+^ and Na^+^, leading to a positive shift in cell resting membrane potential and thereby enabling faster membrane potential kinetics. HCN channels were also recently linked to fast electrical reactivity of fast-spiking cells in the human neocortex [14].

PV neurons also express high levels of P/Q-type calcium channels, which contribute to the fast and precise release of GABA from PV axon terminals into the synaptic cleft [5, 15, 16]. Furthermore, the presynaptic calcium sensor synaptotagmin 2, which is predominantly found in PV neurons and exhibits the fastest calcium-binding kinetics of all synaptotagmins, is thought to also contribute to fast GABA release from PV neurons [17, 18]. Another distinctive feature of PV interneurons is their activity-dependent myelination, as other local interneurons are rarely myelinated [19]. This was recently shown to increase AP conduction velocity and facilitate feedforward inhibition in the cortex [20, 21]. Finally, fast-spiking PV neurons are the only interneurons reported to have autapses, synapses made by a neuron onto itself, both in humans and mice. These autapses have been suggested to increase reliability of fast inhibition by PV neurons and to allow modulation of network activity, and they have been reported in various brain regions [22–24]. In conclusion, unique molecular adaptations allow PV neurons to rapidly transmit electrical and chemical signals and control network activity in a very precise manner.

### Maturation of synaptic contact and firing properties

**Fig. 2:**
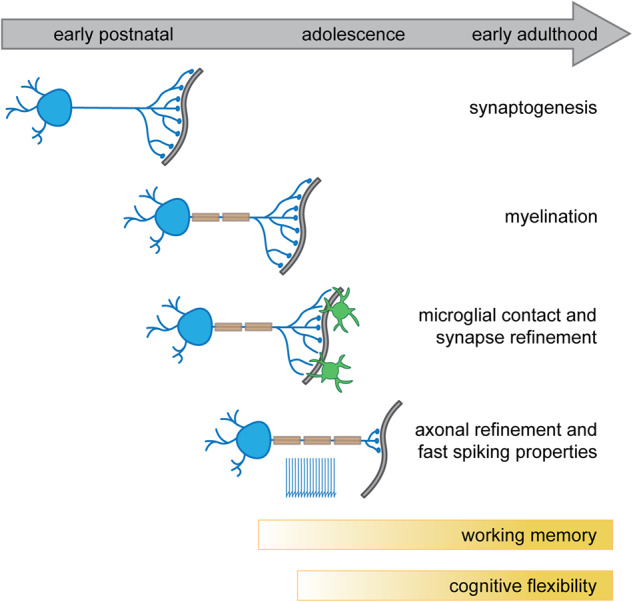
Timeline of major events in the development and maturation of PV interneurons. Synaptogenesis and myelination occur in early postnatal development. Microglia subsequently shape inhibitory synapses and strengthen connectivity between PV neurons and their postsynaptic targets. Next, PV neuron axons are further refined and become fully myelinated, and there is increased expression of ion channels required for fast spiking, enabling PV neurons to achieve precise temporal and spatial control of microcircuits. This postnatal pattern of PV neuron maturation is causally linked to an increase in working memory and cognitive flexibility that is observed until early adulthood.

PV neurons only reach their full electrophysiological and morphological maturation after a defined critical juvenile period (Fig. 2). Following myelination and the appearance of synaptic contacts in the first postnatal week, extensive refinement of PV neuron inhibitory synapses is observed during the weeks thereafter [25, 26]. PV axons display remodeling, including the removal of distal axonal branches and an increase in uniform axonal path lengths and myelination [26]. Studies have suggested that inhibitory PV synapse maturation and refinement depend on critical communication between microglial cells, PV neurons and their post-synaptic targets [27, 28]. In fact, removal of GABA-B1 receptors from microglia during development lead to a decrease in inhibitory synapses in adulthood and impaired GABAergic transmission in the somatosensory cortex of mice [25]. Also, microglia were found to be necessary for bouton maintenance on PV-expressing axo-axonic cells [29]. Moreover, the unique firing properties of PV neurons develop in an activity-dependent manner [30, 31] and presynaptic input onto PV neurons is critical for their maturation and functional integration into networks [32, 33]. The developmental refinement of PV neuron connectivity underlies important mechanisms of learning and plasticity that persist into adulthood [34–38]. Specifically, PV neuron maturation state has been associated with critical period-type plasticity [34–36, 39–41].

Full electrophysiological maturation of PV neurons further depends on the extent of expression of the above-mentioned ion channels. Kv3 channels, for instance, double their expression level from P18 to P30 [42]. Importantly, between postnatal week 2 and 4, PV neurons reduce their input resistance, resting membrane potential, AP duration and latency, and AP propagation time and release period by more than 50%, while rheobase (i.e., the minimum amount of current required to induce AP firing), sag ratio (i.e., the ratio between the steady state decrease in the voltage and the largest decrease in voltage following a hyperpolarizing current step) and firing frequency are drastically increased [42, 43]. Notably, aberrant PV neuron maturation has been suggested as a key player in the pathogenesis of neurodevelopmental disorders such as schizophrenia (see review [19]).

### Control of microcircuits and E/I balance

In addition to their specific molecular attributes, PV interneurons also have distinctive morphological features that allow them to control microcircuits. Their long dendrites cross multiple layers in the hippocampus and in the cortex [2] and they receive excitatory input from many different areas, such as from entorhinal cortex and medial septum in the hippocampus or from thalamic inputs in the cortex, as well as from a vast number of local excitatory neurons across different hippocampal areas or cortical layers [1, 44–47]. While pyramidal neurons in the hippocampus have on average 1.6 spines/μm, PV neurons receive 3.3 excitatory inputs/μm of dendritic shaft [48]. In total, PV neurons receive ten times more excitatory than inhibitory inputs. Conversely, paired recordings from PV and pyramidal neurons revealed that a single PV interneuron projects to almost all local pyramidal neurons within its vicinity [49]. In the hippocampus, one PV neuron contacts approximately 1100 pyramidal neurons with on average six synaptic contacts per neuron [48]. These very specific input and output characteristics allow PV neurons to provide precise feedforward and feedback inhibitory control in hippocampal and cortical microcircuits (Fig. 1A). Accordingly, PV interneurons are crucial for the maintenance of excitation/inhibition (E/I) balance [11, 50–56]. In addition to controlling homeostatic balance at baseline, the precise firing of pyramidal neurons following sensory stimulation also depends on PV neuron-mediated feed-forward inhibition [50, 57–59]. Hippocampal place cell activity, for instance, is generated through an interaction between dendritic excitation by pyramidal cells and perisomatic inhibition by PV interneurons, and optogenetic silencing of PV interneurons increases the firing rate of pyramidal neurons in their place fields during behavior [60]. Entorhinal cortex stellate excitatory cells were shown to be mainly connected via PV interneurons, and this inhibitory microcircuit was found to shape grid-cell firing patterns [61]. By dynamically controlling the firing activity of excitatory cells, PV neurons also play a role in defining the exact population size of pyramidal neurons activated in a specific circuit [37, 62, 63]. This property of PV neurons seems indispensable in the recruitment of defined neuronal ensembles that underlie memory formation and storage (further discussed below).

### Control of network synchrony and generation of network oscillations

Given their ability to control microcircuits and reliably fire at high frequencies, PV neurons play a crucial role in network synchrony and the generation of network oscillations (Fig. 1B, C). Many studies have demonstrated that PV neurons are necessary for the generation of gamma oscillations [22, 50, 64–67] (see review [68]), which range from 30 to 120 Hz and are important for information processing and memory formation [69]. Ex vivo slice and whole-cell recordings demonstrated that PV interneuron-mediated inhibition is crucial to generate gamma rhythms [68, 70]. Recently, autaptic inhibition was shown to facilitate the tuning of PV neuron firing to gamma oscillations [22]. In vivo recordings of neuronal activity further showed that PV neuron APs are phase-locked to gamma oscillations [1, 65, 71], and optogenetic manipulation of PV neurons significantly modulates gamma oscillations [72–74].

In vivo experiments have also demonstrated that PV neuron activity is important for theta oscillations and hippocampal sharp-wave ripple activity, which are both synchronized with cortical oscillations named spindles (12–15 Hz) and are important for memory consolidation [67, 75–78]. Royer et al. found that PV interneurons fire maximally during sharp-wave ripples and preferentially before the trough of the theta wave in head-fixed mice during treadmill running, and that optogenetic silencing of PV interneurons impaired the spike-theta phase relationship of pyramidal neurons during behavior [60]. Optogenetic inhibition of CA1 PV neurons was also shown to disrupt the phase-locking and firing coherence of pyramidal neurons during ripples in CA1 [59]. Optogenetic stimulation of PV neurons, on the other hand, increased theta coherence as well as the firing rate of pyramidal cells during theta oscillations [59, 74, 79]. Stark et al. activated PV interneurons in the hippocampus and neocortex of freely behaving mice and noted that PV neuron activation induced theta-band-limited spiking in pyramidal cells, providing direct evidence for the role of active PV interneurons in theta frequency-favored spiking in these cells [46, 59]. On a network level, chemogenetic inhibition of PV neurons in either CA1 or mPFC blocked the increase in ripple-spindle coupling between these two regions, a phenomenon known to be important in memory consolidation, and prevented the increase in sleep-associated delta, theta and ripple CA1 LFP spectral power [67, 78]. Selective removal of the GABA-A receptor γ2 subunit from PV neurons, which impairs GABA-A receptors function and accordingly fast synaptic inhibition onto PV neurons, was also found to impair theta oscillations and theta-gamma coupling in the CA1, confirming that these network properties critically depend on synaptic inhibition of PV neurons [80]. More recently, PV neuron myelination was shown to significantly contribute to the synchrony of cortical oscillations and the behavioral state-dependent modulation of network activity [20].

## PV neurons in plasticity, learning and memory

### Learning and memory

In view of the importance of PV neurons in network synchrony and in the precise control of microcircuits, their functional involvement in learning and memory is not surprising. Indeed, removal of PV interneurons selectively from the hippocampal CA1 area disrupts spatial working memory [81], and impairing excitatory inputs onto PV neurons has profound effects on learning and memory and associated network alterations [73, 82, 83]. PV interneurons also play a key role in aversive and appetitive associative memory paradigms across brain regions [62, 84–89]. More recently it was shown that when hippocampal PV neuron activity is inhibited during sleep directly following fear-conditioning (FC), memory consolidation is blocked and fear memory impaired [67, 75, 78]. Furthermore, inhibition of PV neurons in the mPFC after FC disrupted long-term fear memory consolidation by impairing ripple-spindle coupling between the hippocampus and the PFC. Inhibiting PV neurons after consolidation or during retrieval had no effect on fear memory [78].

**Fig. 3:**
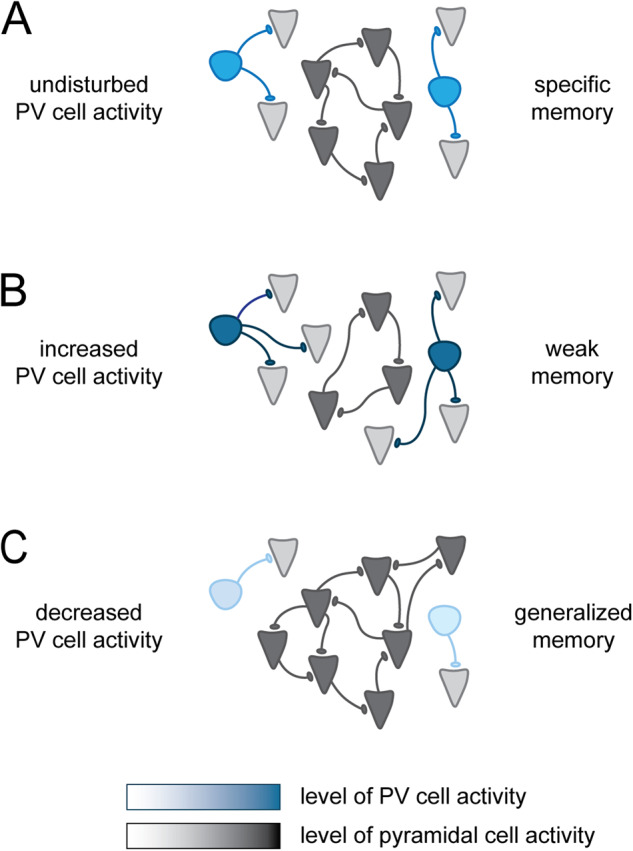
A potential role for PV neurons in shaping memory-specific neuronal ensembles. **A** PV neurons shape memory engrams by preventing excessive activation of pyramidal cells. They thus control the size of an engram and contribute to the storage and recall of specific memories. **B** When PV neuron activity is increased, pyramidal cells receive too much inhibition during memory allocation, and smaller engrams are formed. This would hypothetically result in weaker memories. **C** When PV neuron activity is decreased, too many pyramidal cells are recruited in memory engrams, which would hypothetically lead to larger engrams and generalization of memories. It is important to note that in addition to PV cells, other interneurons have also been shown to play a crucial role in shaping neuronal memory ensembles (see main text for details).

As neuronal excitability determines the recruitment of pyramidal neurons to memory ensembles or engrams [90], PV neurons are likely to also play a role in controlling which neurons are recruited to these ensembles and which are not (Fig. 3). It is hypothesized that PV neurons shape neuronal ensembles by preventing undesired activation of excitatory neurons (see review [91]). Thereby, they may be involved in controlling the size of an engram or its reactivation, and thus contribute to the storage and recall of specific memories [38, 67, 92, 93]. Morrison and colleagues found that silencing PV neurons in the lateral amygdala during FC increases the size of the engram, as indicated by the expression of the activity-dependent gene Arc [94]. PV interneurons are able, via their global and local inhibitory connections, to both shape neuronal ensembles and control the firing rate of neurons involved in such ensembles [92–95]. PV neurons also regulate the spatial tuning of place cells and the maintenance of place fields [96]. Taken together, multiple roles of PV interneurons in contextual memory consolidation have been described. PV cells control the firing rate of excitatory neurons during memory consolidation, shape theta and gamma oscillations, and ensure that only context-specific activities are retained in a local ensemble. It is important to note that other interneurons have also been suggested to control the size of neuronal ensembles involved in memory processing. Somatostatin (SST)-positive interneurons in the hippocampus are activated following FC and suppress surrounding dentate gyrus granule cell activity [97, 98]. Inhibiting SST neurons in the dentate gyrus during FC training enhanced freezing behavior during recall and increased the number of cFos-positive granule cells. Conversely, activation of SST neurons during training led to an impairment in FC memory and a reduced number of cFos-positive granule cells compared to control mice.

### PV neuron plasticity and perineuronal nets

Donato and colleagues put forward a mechanism of PV neuron plasticity by which PV neuron activity is regulated through learning. They showed that PV expression levels in PV neurons correlate with GAD67 levels, and thus may reflect GABAergic output. Environmental enrichment as well as Morris water maze training induced an increase in the relative proportion of what they defined as low-PV neurons, i.e., late-born PV neurons that are marked by relatively low levels of PV. These low-PV neurons also showed an increase in plasticity as measured by a higher turn-over of synapses onto PV neurons and more inhibitory synaptic puncta. Accordingly, chemogenetic inhibition of PV neurons also enhanced plasticity and learning and improved memory formation in familiar object recognition and Morris water maze tasks. On the other hand, contextual FC increased the relative proportion of high-PV neurons, i.e., early-born PV neurons that express relatively high levels of PV, and decreased PV neuron plasticity. Chemogenetic activation of PV neurons disrupted learning and memory in familiar object recognition and Morris water maze tasks by impairing PV plasticity [34, 35] (see review [99]). Interestingly, using chondroitinase ABC, an enzyme that digests chondroitin sulfate proteoglycans in perineuronal nets (PNNs), the authors were able to induce a shift towards a low-PV state [35]. PNNs are net-like structures around specific types of neurons formed by extracellular matrix (ECM) proteins. They are critically involved in brain development and may also control plasticity in the adult brain [100]. In the cortex and the CA1 region of the hippocampus, PNNs are predominantly found around PV interneurons. They have been extensively studied in the visual cortex, where they are necessary for the onset and end of a critical period of brain development [100]. Many studies have now shown that PNNs can affect PV neuron activity and plasticity, and in turn play an important role in learning and memory (see reviews [100, 101]).

## Pv interneuron dysfunction in AD

PV neuron dysfunction has been extensively linked to brain diseases that involve network alterations, decreased plasticity and memory impairment, including Alzheimer’s disease (AD). AD is marked by memory impairment and aberrant neuronal network activity, including alterations in the activation and deactivation of specific brain regions, impairments in oscillatory rhythmic activity and network hypersynchrony. Recent studies show that these changes in network activity are causally linked to memory deficits in AD. Importantly, in AD patients as well as in animal models of AD, restoring network function can improve cognitive abilities, indicating that network imbalance and its underlying neuronal mechanisms, including the involvement of PV neurons, provide attractive substrates for clinical intervention.

### GABAergic dysfunction in AD

APOE4 expression and amyloid-beta (Aβ) accumulation, the former being an important risk factor and the latter a classical hallmark of AD, have both been linked to impaired GABAergic transmission and E/I imbalance, causing hyperexcitability of neuronal networks [102–105]. (For an excellent review on risk factors and protein aggregation hallmarks in AD we refer the reader to [106]). AD patients have an increased incidence and prevalence of seizures (15% and 21% respectively), which is seven times higher than people without dementia [107, 108]. Interestingly, epileptiform activity has also been linked to increased Aβ production and the accelerated deposition of amyloid plaques, hence contributing to AD progression [109, 110]. Subtle changes in hippocampal network activity during the preclinical stages of AD have been proposed in many studies as an early biomarker for the disease [111].

In line with these findings, studies in mouse models of AD have demonstrated that increased expression of amyloid precursor protein (APP) or APOE4 and accumulation of Aβ or tau, all can cause network hypersynchrony (i.e., an aberrant increase in network activity), spontaneous epileptiform activity and inhibitory dysfunction [69, 110]. Palop and colleagues first reported a compensatory remodeling of inhibitory hippocampal circuits as a consequence of aberrant excitation and hypersynchrony [112]. In a follow-up study, they showed that hypersynchrony and epileptiform activity are caused by inhibitory neuron dysfunction [113]. Several studies then confirmed that GABAergic dysfunction is at the core of neuronal network impairment in AD [102, 105, 114–118]. Busche and colleagues reported brain region connectivity disturbances in APP23xPS45 transgenic mice, in particular disturbed long-range coherence among distant cortical areas and cortico-hippocampal and thalamo-cortical areas. Interestingly, they showed that these disturbances are due to impaired GABAergic transmission leading to E/I imbalance. Increasing GABAergic transmission using benzodiazepam treatment, but not decreasing excitatory transmission by blocking glutamate receptors, restored long-range coherence and network dynamics in these mice [119]. Together, these findings provide evidence of a hyperactive neuronal circuitry in AD that is caused primarily by GABAergic dysfunction (see review [102]).

### Impairment of neuronal oscillations in AD

As discussed above, E/I balance is crucial for network oscillations and a loss of inhibitory control can shift E/I balance and induce significant changes in theta and gamma oscillations. Accordingly, various studies observed alterations in oscillatory activity in AD patients in the theta [120–122] and gamma ranges [123–127]. It is important to note that learning has been associated with changes in gamma oscillations and the subsequent encoding of memories has been linked to an increase in gamma power [128–130]. Gamma power in the hippocampus and the frontal cortex decrease with normal aging, suggesting that they are an important substrate for age-related cognitive decline in general [131]. Furthermore, EEG and MEG measurements have revealed AD-associated alterations of brain oscillatory activity at other frequencies, i.e., delta, alpha and beta [132]. Additional studies have reported alterations in hippocampal network activation in AD patients, as well as an increased activation of the default mode network (DMN), known to be involved in high-level cognitive function, during memory recall [69]. Changes in DNM activation also occur during healthy aging as well as in populations at risk for AD, including people with mild cognitive impairment (MCI) and APOE-4 carriers [133]. As such, alterations in hippocampal and cortical oscillatory activity have been proposed as early biomarkers of AD [111, 134]. Notably, all AD mouse models to our knowledge present, to different degrees, alterations in network oscillations and many studies have confirmed specific changes in gamma and theta oscillations in vivo and ex vivo (Table 1), making oscillatory network changes one of the most robust endophenotypes in mouse models of AD that matches clinical observations in humans.

**Table 1:** Altered neuronal oscillations and PV neuron impairments in mouse models of AD.

AD model	Oscillatory changes	PV neuron dysfunction	Rescue experiments
hAPP	◄ Gamma [113, 147, 149] ◄ Theta (ex vivo) [211]	◄ Functional impairment of PV neurons [113, 147, 149] ◄ PV neuron excitability decreased [211]	◄ Nav1.1 overexpression [113] ◄ 40 Hz PV stimulation [147] ◄ MGE-derived neuron transplant [149] ◄ Erb4 deletion from PV neurons [212]
5xFAD	◄ Gamma [148]	◄ Functional impairment of PV neurons [148] ◄ Decreased PV expression [213, 214]	◄ 40 Hz PV stimulation [148]
APP/PS1	◄ Gamma [154] ◄ Theta [215] ◄ Theta and Gamma [216] ◄ Gamma (ex vivo) [217]	◄ Functional impairment of PV neurons [142] ◄ Decreased PV expression [150, 217, 218]	◄ Restoring PV activity [142] ◄ Restoring GABAergic activity [216] ◄ Transplantation of GABAergic interneuron progenitor [150] ◄ Rescue with Citalopram [218]
TgCRND8	◄ Gamma [219] ◄ Theta and Gamma (ex vivo) [220]	◄ Decreased PV expression [135, 138, 139, 219]	◄ γ-secretase (semagacestat) inhibition recued Nav1.1 levels and spatial memory[219]
P301L	◄ Theta and Gamma [221]	◄ Decreased PV expression [167]	◄ Gamma entrainment [222, 223]
Tg2576	◄ Theta [224, 225]	◄ Input onto PV [151] ◄ Decreased PV expression [136, 137]	◄ EE [136] ◄ Optogenetic activation of ECIIPN–CA1PV synapses with a theta burst stimulation [151]
App KI	◄ Gamma [226]	◄ Functional impairment ◄ Decreased PV expression [140]	◄ GABA agonist [140]
APP(SL)/PS1 KI	N/A	◄ Decreased PV expression [141]	N/A
rTg4510	◄ Theta [227] ◄ Gamma (ex vivo) [228] ◄ High-frequency ripple oscillations [227, 229]	◄ No change in PV expression [228]	N/A
TAS10	◄ Gamma (ex vivo) [230]	N/A	N/A
3xTg-AD	N/A	◄ Decreased PV expression [231]	N/A
VLW	N/A	◄ Input onto PV [232]	N/A
TauPS2APP	N/A	◄ Decreased PV expression [233]	N/A
App/PS1-CB1−/−	N/A	◄ Decreased PV expression [234]	N/A
APP/PS1/Nptx2−/−	N/A	◄ PV output onto PYR [235]	N/A
Aβ application	◄ Theta [236] ◄ Gamma (ex vivo) [237–239] ◄ Theta (ex vivo) [240, 241]	◄ PV output onto PYR [153] ◄ Decreased PV expression [242]	N/A

### Impaired PV neuron activity in AD

Since many studies have placed PV neurons at the center of network oscillations and memory processes, as described in the sections above, it is not surprising that the observed changes in AD patients and mouse models, including changes in gamma and theta oscillations and cognitive deficits, are potentially linked to PV neuron dysfunction. Verret and colleagues were the first to show that PV neurons are impaired in hAPP mice. This was accompanied by alterations in gamma power and increased epileptic activity. Interestingly, the authors demonstrated that PV neurons show decreased expression of the Nav1.1 sodium channel subunit and reported a similar decrease in post-mortem brain tissue of AD patients. Overexpressing Nav1.1 in hAPP mice rescued network imbalance, gamma power and memory impairment in these mice [113]. In addition, a significant decrease in the number of PV neurons, as measured by a decrease in PV levels, has been reported in several AD mouse lines [135–141], although several other studies have reported no alterations in PV neuron numbers [142–146]. Given the fact that restoring GABAergic transmission, via either chemogenetics, optogenetics, genetic manipulations or even interneuron transplants, can rescue network function and behavior [82, 113, 119, 140, 142, 147–151], the reported decrease in PV neurons number may reflect a decrease in PV expression rather than an actual loss of neurons. Additional studies exploring the correlation between PV expression and GABAergic activity in AD and investigating PV neuron survival are necessary to test whether PV neurons are more prone to neuronal loss in AD. One consistent finding among all these studies, however, is a decrease in inhibitory transmission and activity in AD that is likely due to a specific impairment of PV neurons, even though other interneurons may also be contributing to this dysfunction [98, 152, 153], resulting in a failure of inhibitory control, oscillatory changes, an overall increased excitation and epileptic activity, and cognitive decline.

### Restoring or mimicking PV neuron function in AD

In the last decade, many studies have focused on restoring PV neuron function in order to rescue AD pathology and cognitive impairment (Table 1). Data from our lab showed that restoring PV neuron activity in the hippocampus of APP/PS1 mice has beneficial effects on hippocampal networks and spatial memory on the short and long-term. Iaccarino and colleagues showed that in 5xFAD mice, gamma activity is significantly lower than in wild type control mice. Stimulating PV interneurons optogenetically at 40 Hz for 1 h restored gamma activity and led to a significant microglia-mediated decrease in soluble and insoluble Aβ levels, hence delaying AD pathology [148]. Moreover, other studies have validated that either direct or indirect stimulation of PV interneurons can restore theta and gamma oscillations as well as memory performance in different AD mouse models, as summarized in Table 1. Taken together, these studies provide a strong case for PV neuron hypofunction being causally involved in network and memory problems in AD and providing a substrate for non-invasive interventions.

## Mechanisms of PV neuron dysfunction in AD

While the above studies highlight PV neurons as an attractive and highly relevant target for restoring network function and memory in AD, an important question remains whether PV neuron impairment is a cause or a consequence of AD pathogenesis. One possibility is that PV neuron dysfunction is due to Aβ-dependent intrinsic changes in channel properties or expression. Alternatively, PV neurons may become impaired because of changes in afferent synaptic inputs and impaired synaptic transmission onto PV neurons, or due to a high vulnerability in general resulting from their above-mentioned unique fast-spiking properties. Further insight into the earliest PV neuron alterations in AD might help to shed light on this issue.

### Early and transient hyperexcitability of PV neurons

In a recent series of experiments from our lab we investigated PV neuron intrinsic properties and associated microcircuit adaptations in the hippocampus of APP/PS1 mice at an early disease stage, i.e., at 3 months of age, when memory deficits are first detected. We showed that hippocampal PV neurons, but not pyramidal neurons, are initially and transiently hyperexcitable at this age. At a later disease stage, i.e., at 7 months of age, PV neurons appeared to be hypoactive while pyramidal neurons were hyperexcitable. Importantly, inhibiting PV neuron activity at an early stage or stimulating PV neuron activity at a later stage, both restored Morris water maze performance in APP/PS1 mice, suggesting that both states, hyperexcitable first and hypoexcitable later, are causally linked to memory impairment in AD [142]. [140]Interestingly, early hyperexcitability of PV neurons is paralleled by an increase in PNNs surrounding PV cells, which may serve to support the increased energy demand of these cells, but at the same time also limits plasticity and causes memory impairment [146]. Together, these findings suggest biphasic alterations in PV neuron activity during amyloidosis in APP/PS1 mice and potentially different mechanisms of memory impairment at different disease stages. Interestingly, biphasic alterations in inhibitory transmission were also reported in other studies [154, 155] and biphasic alterations in network connectivity have been observed during AD progression in patients [156, 157]. It would be relevant to test whether an aberrant early increase in PV neuron activity contributes to the silencing of the (prospective) engram neurons and impairing memory retrieval (Fig. 3). In fact, Roy et al. showed a decrease in FC-activated excitatory engram neurons in the dentate gyrus of AD mice. Optogenetic reactivation of these cells restored fear memory in AD mice [158]. Future studies could investigate whether silencing of PV neurons in the dentate gyrus could also restore fear memory in these mice.

**Fig. 4:**
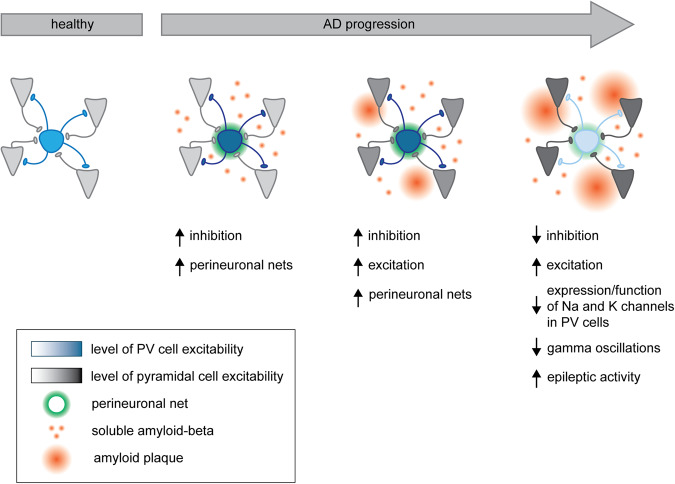
A hypothetical pathogenic mechanism for neuronal network changes in AD based on PV neuron dysfunction. Neuronal hyperexcitability and network dysfunction are commonly observed in AD patients and in mouse models of AD. Taken together, published data seem to support a model in which interneurons, in particular PV interneurons, play a key role. Early in disease pathogenesis, before any significant aggregation of Aβ into plaques, soluble Aβ causes an aberrant increase in the excitability of PV cells and a corresponding increase in network inhibition. This is accompanied by an increase in perineuronal nets around PV cells, possibly to support the increased energy demand due to higher activity. Pyramidal cells at this stage seem unaffected, possibly because they are less vulnerable or less sensitive to Aβ toxicity. At a later stage, pyramidal cells also show increased excitability, possibly as a homeostatic response to restore excitation/inhibition balance. Increasing Aβ concentrations and Aβ aggregation may contribute to the increased excitability of pyramidal cells. Finally, PV cells become hypoactive while pyramidal cells remain hyperexcitable due to increasing Aβ pathology, resulting in an overall network hyperexcitability. The reduced activity of PV neurons is linked to a reduced expression and/or function of Nav1.1 and Kv3 channels. This stage is supported by both patient and mouse data showing reduced gamma oscillations and increased epileptic activity.

To test whether early PV neuron hyperexcitability causally contributes to AD pathogenesis, we artificially induced PV neuron hyperexcitability in wild type mice and used selective chemogenetic activation to trigger a persistent AD-like hippocampal network state [159]. Under these conditions PV neurons not only became persistently hyperexcitable, but they were also more sensitive to a low dose of amyloid-beta (Ab) injected directly into the hippocampus. While infusing the same concentration of Aβ in healthy mice had no cellular nor behavioral effect, under conditions of induced PV neuron hyperexcitability, this low concentration of Aβ was able to cause PV neuron hypo-excitability, increase pyramidal neuron firing frequency, disrupt synaptic transmission onto pyramidal neurons and significantly impair spatial memory. These findings suggest that early PV neuron hyperexcitability could be a first and causal step in later PV neuron hypofunction, but only under AD-like conditions of increased Aβ levels, which may be a key mechanism in triggering network and memory impairments in AD. Given the biphasic nature of PV neuron dysfunction in APP/PS1 mice, we hypothesize that the reported decrease in PV expression and reduced activity of PV neurons observed at later stages in various AD mouse models are a consequence of early PV neuron hyperexcitability (Fig. 4). Indeed, reducing PV neuron activity at an early stage, using selective chemogenetic, inhibition restored synaptic transmission and intrinsic properties of both PV neurons and pyramidal neurons as well as spatial memory up to eight weeks after the end of the intervention [142].

PV neuron hyperexcitability may also contribute to amyloid pathology, as early restoration of PV neuron activity led to a decrease in amyloid pathology, and blocking Aβ production specifically in GABAergic neurons significantly reduced plaque pathology [142, 148, 160]. Indeed, APP is prominently expressed in a subset of hippocampal interneurons; 53% of PV cells in the CA1 region of the hippocampus are APP-positive and hyperphosphorylated tau as well as Aβ were found to accumulate in PV neurons in different AD mouse models [160–162].

### Selective vulnerability of PV neurons and associated microcircuits

The observation that early PV neuron hyperexcitability is causal to several AD-like cellular, network and behavioral adaptations is intriguing, but a question that remains is what elicits PV neuron hyperexcitability in the first place? One possibility is that these neurons are more vulnerable to Ab-induced stress than other neurons. Treating APP/PS1 mice early with a β-site APP cleaving enzyme 1 (BACE1) inhibitor completely restored PV neuron excitability and function, and when hippocampal slices from wild type mice were exposed to Aβ ex vivo, PV neurons quickly became hyperexcitable while pyramidal neurons did not [142]. Moreover, Aβ significantly impaired PV neuron function in an induced-PV hyperexcitability model [159]. Multiples studies have highlighted an increased vulnerability of PV neurons to Aβ toxicity (Table 1), suggesting that PV neuron dysfunction in AD is, at least in part, Ab-dependent. Little is known about the direct molecular interactions of Aβ with receptors or channels on GABAergic neurons, or on PV neurons specifically. As mentioned previously, PV neurons express specific ion channel subunits, such as Nav1.1 and Kv3, which are required for their fast-spiking properties. Interestingly, BACE1 expression is linked to Nav1.1 expression as BACE1-null mice have reduced Nav1.1 protein levels [163]. Studies have also demonstrated that Aβ affects various synaptic inputs from and onto PV neurons [164, 165]. Exploring further the direct effect of Aβ on PV neuron channels and synapses would help elucidate the mechanisms underlying PV neuron vulnerability in AD. The combined observation of early Ab-induced PV neuron hyperexcitability and selective Ab-dependent PV cell vulnerability suggest a pathogenic feedback loop in which increasing Aβ levels cause PV neuron hyperexcitability and hyperactive PV neurons in turn contribute to increased Aβ production.

### An interplay between tau and amyloid?

Tau mouse models also display aberrant network activity and inhibitory network impairments. Tau pathology was found to cause an increase in interneuron activity, grid cell dysfunction and loss of excitatory neurons in mice expressing tau-P301L in the entorhinal cortex. This was accompanied by an enhancement of theta power and a disruption of spatial memory [166]. In another study, P301L mice showed a loss of interneurons, which was associated with impaired synaptic plasticity as well as cognitive deficits. Moreover, a GABA-A receptor agonist was effective in restoring both the cognitive and synaptic impairments, supporting the role of inhibitory neurons in tau-dependent neuronal network dysfunction [167]. The question remains if these changes in interneuron function reflect a direct effect of tau toxicity or network adaptations in response to tau pathology.

Interestingly, Aβ seems to play a role in tau pathology. Aβ was shown to increase tau spreading and toxicity in a study where rTgTauEC mice were crossed with APP/PS1 mice [168]. Several studies have confirmed that tau and Aβ pathology are usually comorbid [169, 170]. A recent study by Busche et al. puts forward a new mechanism of network dysfunction where Aβ promotes neuronal hyperactivity while tau suppresses activity. Whereas in APP/PS1 mice a significant percentage of neurons were clearly hyperactive, in rTg4510 mice many neurons were silent. When the two lines were crossed, tau blocked Ab-dependent hyperactivity, resulting in mainly silent neurons. Intriguingly, when the authors suppressed tau expression, they could rescue circuit activity in tau mice but not in mice with both tau and Aβ pathology [171]. As Aβ is undeniably involved in neuronal hyperactivity, it might be that an early Ab-dependent hyperactivity of PV neurons paves the way to an imbalanced activity and circuit dysfunction, which worsens with increasing tau levels and eventually leads to neurodegeneration.

### Glial cells and inhibitory dysfunction

Glial cells have been at the center of some recent AD studies [172–174]. Their involvement may go beyond a simple response to protein aggregation and neurodegeneration, as their roles in maintaining E/I balance [175], synaptic plasticity [176], network activity [177] and memory processing [178, 179] have been firmly established. However, as regulators of neuronal network balance and synaptic transmission, their contribution to AD needs further exploration.

Microglia are thought to be important for Aβ clearance [180–183]. Activated microglia are present in close proximity of amyloid plaques [184–186]. Genome-wide association studies for AD have up to now identified 75 risk loci for AD and pathway enrichment analysis of risk genes clearly confirmed microglial involvement [187]. For instance, a mutation in the triggering receptor expressed on myeloid cells 2 (TREM2), a receptor specific to microglia, significantly increases risk for AD [47, 188]. Importantly, this mutation was demonstrated to reduce the role of TREM2 in the activation of microglia, hence leading to reduced Aβ clearance [189, 190]. In a study by Iaccarino et al., optogenetic 40 Hz stimulation of PV neurons induced morphological changes in microglia of 5xFAD mice and reduced the levels of both soluble and insoluble Aβ [148]. This suggests that PV neuron activity is causally linked to microglia activation and Aβ clearance. The mechanism behind this link remains unclear, and a remaining question is whether microglia also play a role in regulating PV neuron activity. Several studies have reported a microglia-dependent loss of synapses is early in AD, leading to neurodegeneration and memory loss [191–193]. Moreover, microglia are able to selectively prune inhibitory synapses, although this was only observed during postnatal development [25]. Thus, microglia could be contributing to inhibitory dysfunction and network alterations in AD by weakening inhibitory synapses and through a defective clearance of Aβ around specific synapses of interneurons.

Astrocytes have similarly been implicated in network imbalance in AD because of their role in synaptic, specifically GABAergic, transmission [194–200]. GABA levels in reactive hippocampal astrocytes are increased in AD mouse models and in AD patients [201–203]. It was further revealed, in a mouse model of AD, that GABA released from astrocytes activates GABA-A receptors and impairs LTP induction in the hippocampus. Notably, when astrocytic GABA synthesis or release were inhibited, both LTP and memory were restored [202]. Several other studies have confirmed the role of reactive astrocytes in network imbalance and impaired synaptic plasticity in AD [196, 204]. Interestingly, astrogliosis has been reported at a very early stage of AD [174, 205]. Taken together, network imbalance in AD, and specifically the dysfunction of inhibitory neurons, might in part be due to alterations in astrocyte activity at early disease stages. Further studies are needed to confirm a potential role of astrocytes specifically in PV neuron dysfunction in AD.

Finally, while AD has been mainly considered as a gray matter disorder, recent evidence points to myelin impairment as a potential disease mechanism. It has been proposed that myelin damage might occur earlier than Aβ and tau pathology in AD [206], and restoring myelin deficits in APP/PS1 mice rescues cognition and hippocampal physiology [207]. Interestingly, activity-dependent myelination driven by absence seizures was shown to contribute to epilepsy progression [208]. In a recent study, myelinated axons of PV neurons were found to contain a significant number of clustered mitochondria. Mitochondrial calcium transients were shown to be modulated by myelination on PV axons and myelin loss caused deficits in mitochondrial calcium responses at branch points [209]. These findings highlight an intricate link between myelination and metabolic support that could specifically affect PV cells in the early stages of AD. Whether deficits in PV neuron myelination could also be involved in the increased risk for epilepsy or the cognitive decline observed in AD requires further investigation, especially with the emergence of glial cells as important players in inhibitory dysfunction and network abnormalities.

## Conclusion

PV neuron dysfunction is clearly related to network impairments and cognitive deficits in AD. What makes these neurons so vulnerable? One hypothesis has been that their ability to fire at high frequencies, and the corresponding high energy consumption, contribute to PV neuron vulnerability. However, a recent paper by Hu and Jonas showed that high-frequency firing of PV cells is very efficient. The energy required for one AP was only 1.6 times the theoretical minimum. This efficiency was due to the fast inactivation kinetics of sodium channels combined with a delayed activation of Kv3-type potassium channels in PV neurons [210]. Hence, any alteration in the expression or activity of these channels in AD could make PV neurons vulnerable. In this review, we propose that PV neurons, because of their distinct molecular and morphological characteristics, are selectively vulnerable to Aβ toxicity, and that their selective vulnerability is at the core of early network dysfunction in AD. More studies are needed to investigate how Aβ selectively affects the excitability of PV neurons, and which specific ion channels are involved. Aβ may also impair afferent inputs onto PV neurons, which on the long term could lead to a hypofunction of PV neurons. Finally, specific AD-related genetic risk factors, such as APOE4, may be contributing to early changes in PV neuron excitability in the adult brain, which renders the neuronal circuitry more vulnerable to Aβ toxicity, initiating network dysfunction, hyperactivity of principal networks and cognitive deficits.
